# Case report: an initially unresectable stage III pulmonary sarcomatoid carcinoma qith EGFR mutation achieving pathological complete response following neoadjuvant therapy with osimertinib plus chemotherapy

**DOI:** 10.3389/fonc.2022.1033322

**Published:** 2022-11-25

**Authors:** Xiguang Liu, Yating Zheng, Shijie Mai, Yu Tong, Lili Yang, Mengli Huang, Ruijun Cai

**Affiliations:** ^1^ Nanfang Hospital, Southern Medical University, Guangzhou, China; ^2^ Medical Department, 3D Medicines Inc., Shanghai, China

**Keywords:** pulmonary sarcomatoid carcinoma, osimertinib, chemotherapy, neoadjuvant, EGFR

## Abstract

Epidermal growth factor receptor (EGFR) tyrosine kinase inhibitors (EGFR-TKIs) provide dramatic response to patients with advanced EGFR-mutant non-small cell lung cancer (NSCLC). However, the use of neoadjuvant therapy with EGFR-TKIs in EGFR-mutant NSCLC remains controversial, especially in pulmonary sarcomatoid carcinoma (PSC). One patient with initially unresectable stage III (cT4N0M0) PSC was found to carry EGFR mutation by the next generation sequencing. After neoadjuvant therapy with osimertinib plus chemotherapy, radical resection of the right upper lung lesion was achieved, and the pathological results reached pathological complete response (pCR). To the best of our knowledge, this is the first report of an EGFR-mutant patient with initially unresectable stage III PSC achieved pCR by neoadjuvant therapy with osimertinib plus chemotherapy. Therefore, neoadjuvant therapy with EGFR-TKIs may be a viable option for EGFR-mutant PSC patients.

## Introduction

Pulmonary sarcomatoid carcinoma (PSC) is a rare subclassification of non-small cell lung cancer (NSCLC), accounting for 0.5% (1). Compared with other histological subtypes of NSCLC, including lung adenocarcinoma (LUAD), lung squamous cell carcinoma (LUSC), large cell neuroendocrine carcinoma (LCNEC), and large cell carcinoma (LCC), PSC is more aggressive, has a lower response rate to conventional therapy, and a higher recurrence rate (2–4). It carries a poor prognosis (a 5-year survival of 24.5%) (4), due to early metastasis and high resistance to platinum-based chemotherapy (5, 6). It is well known that the treatment principle of PSC is similar to that of NSCLC, and radical surgery is the recommended treatment for early-stage patients (7). The treatment of stage III patients is highly individualized and guided by multidisciplinary input based on patient factors. Neoadjuvant chemotherapy can be performed in some stage III patients, and surgical resection can be performed if it is considered feasible (8, 9). Although both neoadjuvant and adjuvant chemotherapy improve survival in patients with NSCLC, the effect of perioperative chemotherapy on PSC is controversial, as is radiotherapy (10, 11). Therefore, it is critical to explore new therapeutic targets and therapeutic methods for PSC.

The mutation frequency of epidermal growth factor receptor (EGFR) in PSC is 19% (12). Currently, several EGFR tyrosine kinase inhibitors (EGFR-TKIs) have been approved by the U.S. Food and Drug Administration (FDA) as first-line therapy for advanced NSCLC with EGFR mutations (13), but there is still a lack of convincing evidence for their use as neoadjuvant therapy for stage III NSCLC. Currently, several clinical trials of EGFR-TKIs in the neoadjuvant treatment of NSCLC are underway (**Supplementary Table 1**). In addition, several case reports and small sample size studies have shown that EGFR-TKIs as neoadjuvant therapy for EGFR-mutant stage II-III NSCLC are acceptable in terms of efficacy, safety and surgical complications (14–19), but these reports and studies has not defined the benefit for PSC. To the best of our knowledge, we report the first case of neoadjuvant therapy with osimertinib plus chemotherapy in an initially unresectable stage III PSC.

## Case presentation

An 50-year-old male was admitted to hospital on August 22^th^, 2021 for cough and expectoration without obvious cause. He had 35 years history of smoking a pack a day, and history of drinking 100ml/day. He had no history of infectious diseases such as dysentery, malaria, viral hepatitis or tuberculosis. His father died of esophageal cancer, denying a family history of the disease.

**Figure 1:**
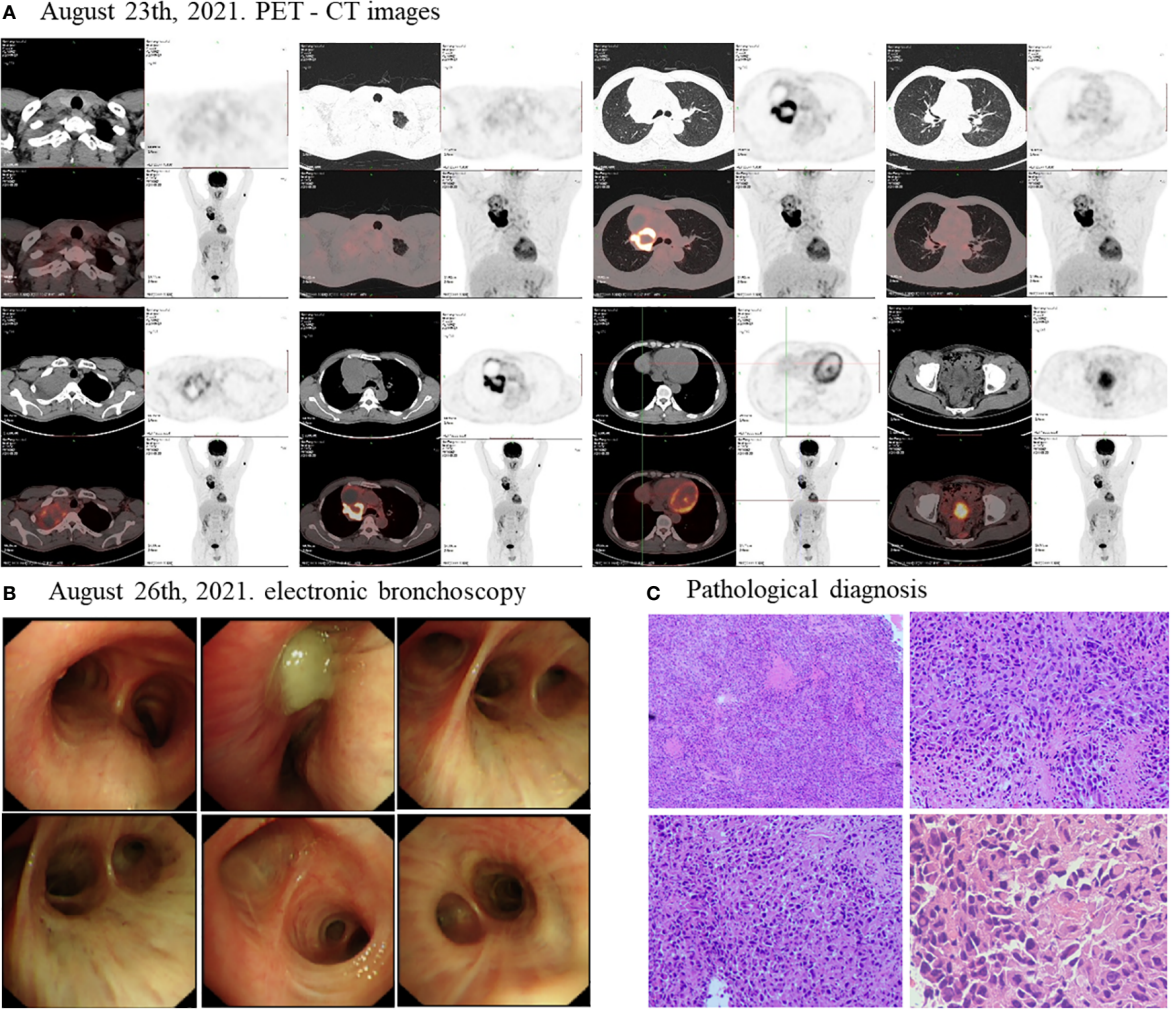
Disease status before treatment. The PET-CT scan **(A)** showed that before treatment, the size of the lung field from the right hilum to the right upper lobe of the lung large cystic solid mass was 8.6 × 5.9 × 9.9 cm. **(B)** The electronic bronchoscopy of the patient before treatment. **(C)** Pathological diagnosis of the patient before treatment.

As the timeline of the clinical course in this patient (**Supplementary Figure 1**), On August 23^th^, 2021, a positron emission tomography-computed tomography (PET-CT) revealed a large cystic solid lesion (8.6 × 5.9 × 9.9 cm) in the lung field from the right hilum to the right upper lobe, which was considered to be a right thoracic malignant tumor. The lesion invaded mediastinum adjacent superior vena cava and main right pulmonary artery. Additionally, multiple enlarged lymph nodes were observed in both hilum, mediastinum and right supraclavicular fossa, with the largest being 1.5×1.4 cm, with mild to moderate increase in metabolism, which was considered as multiple lymph node inflammation (**Figure 1A**). On August 26^th^, 2021, an electronic bronchoscopy revealed stenosis of the right upper lobe opening and obstruction of the lumen by necrotic material (**Figure 1B**). An electronic bronchoscopy biopsy of the superior lobe of right lung led to a pathological diagnosis of PSC (**Figure 1C**). The malignancy was consistent with stage III (cT4N0M0) disease. Upon NGS analysis of the biopsy tissue sample, the patient was identified to harbor EGFR mutations (EGFR exon21 p.L861Q, EGFR exon18 p.G719C) (**Supplementary Figure 2**), TP53 mutation (exon8 p.R273C), BCORL1 amplification, and CCNE1 amplification. In addition, the patient was identified as having microsatellite stable (MSS) and tumor mutation burden (TMB) of 6.15Muts/Mb by NGS. In addition, immunohistochemistry (IHC) for PD-L1 was strong positive (TPS=80%).

**Figure 2:**
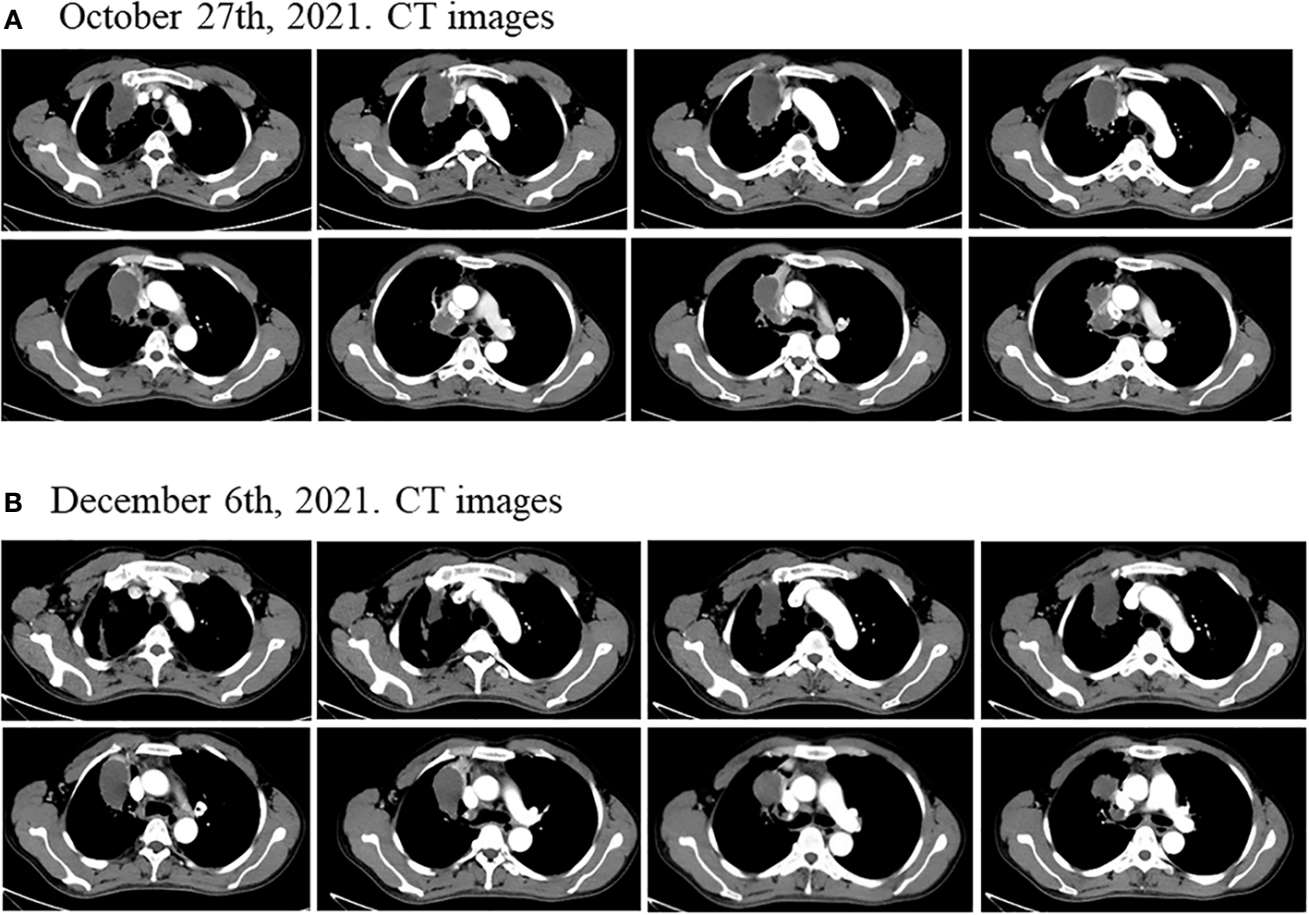
Disease status after neoadjuvant chemotherapy plus immunotherapy and chemotherapy plus EGFR-TKI. **(A)** After two cycles of neoadjuvant chemotherapy plus immunotherapy, the size of the lung field from the right hilum to the right upper lobe of the lung large cystic solid mass was 6.6 × 4.6 × 6.0 cm. **(B)** After one cycle of neoadjuvant chemotherapy plus EGFR-TKI, the size of the lung field from the right hilum to the right upper lobe of the lung large cystic solid mass was 6.2 × 3.5 × 5.5 cm.

**Figure 3:**
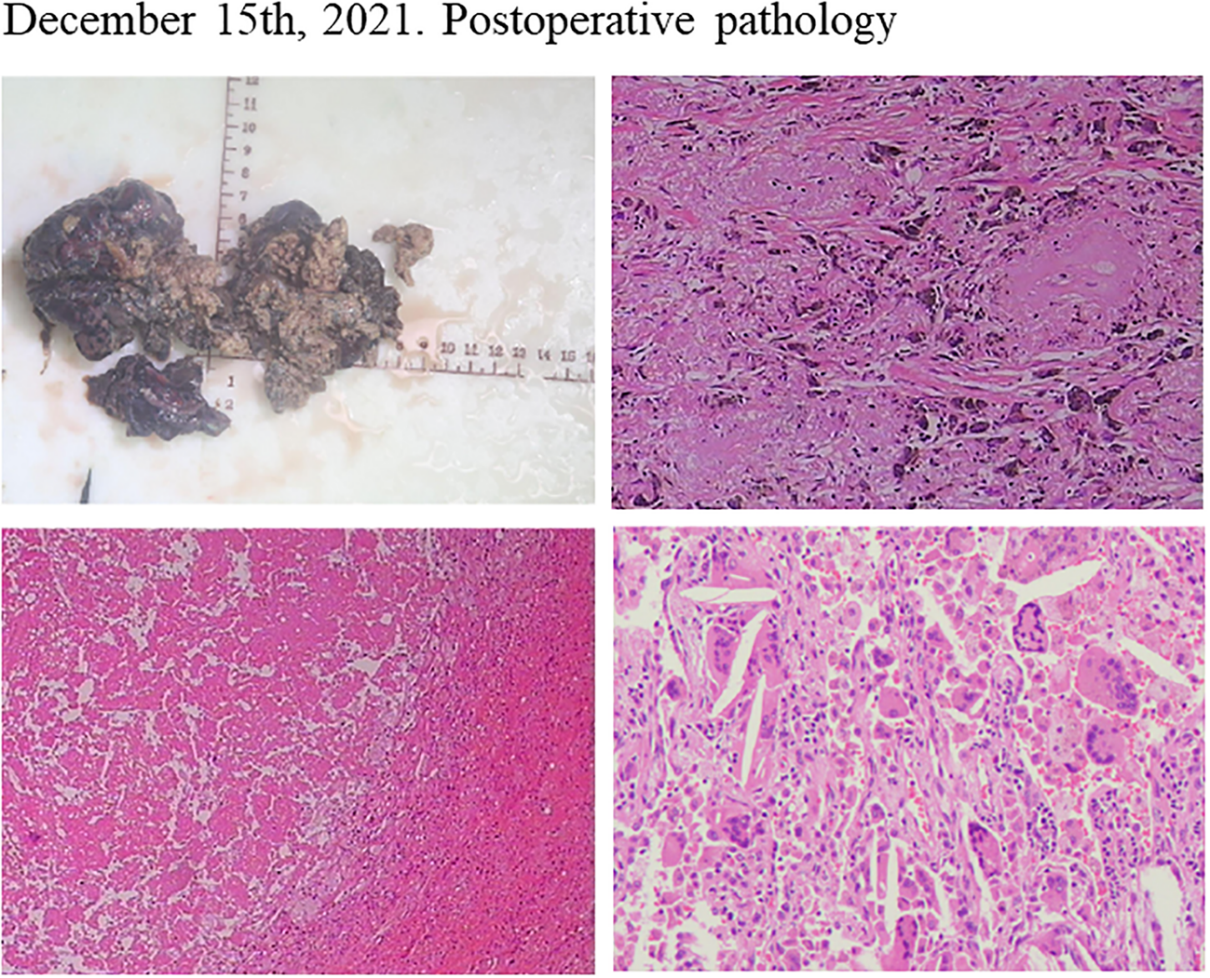
Postoperative pathology of the patient after neoadjuvant chemotherapy plus EGFR-TKI showed pathological complete response.

**Figure 4:**
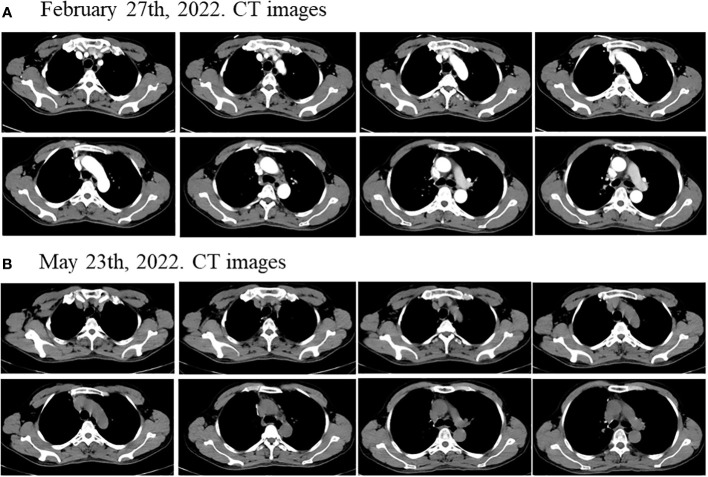
Disease status after surgical resection. The chest CT scan showed no evidence of malignant tumor recurrence about **(A)** 2.5 months and **(B)** 5.5 months after surgical resection.

Considering the patient’s PET-CT results showed that the tumor was large and adjacent to the mediastinum and main pulmonary artery, which made it difficult to resect. The patient had no indications for surgery, and chemotherapy plus immunotherapy (albumin paclitaxel 400mg d1+ cisplatin 90mg dl+ tislelizumab 200mg dl) was subsequently started on September 4^th^ and September 30^th^, 2021. After two cycles of chemotherapy plus immunotherapy, on October 27, 2021, a chest computed tomography (CT) scan showed that the size of the lesion in the lung field from the right hilum to the right upper lobe was 6.6 × 4.6 × 6.0 cm, and the largest enlarged lymph node was 1.5 × 1.3 cm, but the lesion was still close to the mediastinum and the main pulmonary artery, which was difficult to be surgically removed (**Figure 2A**). In addition, the patient’s transaminase increased during the treatment period, indicating abnormal liver function, liver protection treatment returned to normal after a period of time. Moreover, it has been reported that immunotherapy may cause abnormal liver function in NSCLC patients, in which transaminases are increased (20–22). Considering that immunotherapy may cause liver injury, immunization was discontinued. Immediately after, the patient received chemotherapy plus EGFR-TKI (albumin paclitaxel 400mg d1+ cisplatin 50mg dl+ osimertinib 30mg dl-14) on October 30^th^, 2021. On December 6, 2021, after one cycle of chemotherapy plus EGFR-TKI, a chest CT scan showed that the size of the lesion in the lung field from the right hilum to the right upper lobe was 6.6 × 4.6 × 6.0 cm, and the largest enlarged lymph node was 1.7 × 0.8 cm (**Figure 2B**). After chemotherapy plus EGFR-TKI treatment, there was a gap between the lesion and the mediastinum, as well as between the lesion and the right pulmonary trunk, indicating that the lesion may be resected by surgery. Since no obvious contraindications were found, the patient underwent thoracoscopic radical resection of right upper lung cancer on December 15^th^, 2021. Postoperative pathology showed no residual tumor cells in the right upper lung lesion, no residual tumor cells in the resection margins of the trachea and blood vessels, and no tumor metastasis in the lymph nodes, reached pathological complete response (pCR) (**Figure 3**). On February 27^th^ and May 23^th^, 2022, about 2.5 months and 5.5 months after surgical resection, chest CT scan showed no evidence of malignant tumor recurrence (**Figures 4A, B**).

## Discussion

At present, there is no standardized treatment for managing PSC, a rare NSCLC subtype with poor prognosis, and its treatment strategy is derived from NSCLC. The use of EGFR-TKIs has revolutionized the treatment of advanced NSCLC with EGFR mutations. In the perioperative treatment of early NSCLC, ADJUVANT, EVAN and SELECT clinical trials have shown that gefitinib and erlotinib, respectively, as postoperative therapies can improve disease-free survival (DFS) in EGFR-mutant patients (23–25). Similarly, the ADAURA trial also showed that osimertinib as adjuvant therapy significantly improved DFS in patients with stage IB-IIIA EGFR-mutant NSCLC (26). In addition, there are a few case reports and small sample size studies indicating the feasibility of EGFR-TKIs (Gefitinib, Erlotinib, Afatinib, Osimertinib, Aumolertinib) in the neoadjuvant stage of EGFR-mutant NSCLC patients (14–19). But these treatments have not been evaluated in PSC.

In this case, after two cycles of chemotherapy plus immunotherapy, the tumor was still inoperable, and during the treatment, the liver function of the patient was abnormal and the transaminase was elevated. After consulting the relevant literature, it was found that immunotherapy may cause liver function abnormalities in NSCLC patients, so immunotherapy was stopped after comprehensive consideration (20–22). Then the initially unresectable stage III PSC patient received osimertinib plus chemotherapy. The CT images after osimertinib plus chemotherapy treatment showed slight tumor shrinkage and the opportunity for surgery. It is difficult to determine how much of this response is due to the delayed effect of chemotherapy plus immunotherapy or to the effect of osimertinib or osimertinib plus chemotherapy. Despite this, the patient had a good treatment response, with complete resection of the primary tumor, pCR on pathological evaluation, and no disease recurrence 5.5 months after surgery. At present, there are few clinical studies on immunotherapy specifically for PSC. A retrospective study with a small sample size enrolled 37 PSC patients who received anti-PD-1 immunotherapy, and 40.5% of them achieved clinical remission, but OS was not statistically significant. In addition, we reviewed the biomarker analysis of this study and found that 19 patients tested for PD-L1 had a median PD-L1 expression of 70% and only one PD-L1-negative sample. Although the expression of PD-L1 in PSC is generally higher, the results of prognosis analysis of patients showed that there was a trend that the median OS of PD-L1+ group was higher than that of PD-L1- group, but there was no significant statistical significance. Moreover, the expression of PD-L1 in disease progression (PD), stable disease (SD) and partial response (PR) patients did not show an obvious trend of gradual increase (26). Only one patient with a detectable EGFR exon 18 mutation, TP53 mutation and low TMB (4Muts/Mb) showed early progression on immunotherapy, with a treatment duration of 0.4 months and an OS of 1.8 months (26). The patient we reported here also carried EGFR exon18 mutation, combined with TP53 mutation and low TMB (6.15Muts/Mb), which is similar to the above patient, which may imply that EGFR-mutated NSCLC patients, even PSC patients, may have a low benefit from immunotherapy. This case report suggests to some extent that that neoadjuvant therapy with osimertinib plus chemotherapy may be both effective and safe in stage III PSC and neoadjuvant therapy with EGFR-TKIs may be another viable option for patients who do not respond well to other neoadjuvant therapies.

In conclusion, although the expression of PD-L1 in PSC is generally high, and the positive rate is higher than that of other NSCLC, we cannot judge whether the expression of PD-L1 and the level of TMB are significantly positively correlated with the prognosis of immunotherapy in PSC patients based on the few clinical studies and data related to immunotherapy in PSC patients. And whether PSC patients with EGFR mutations benefit less from immunotherapy than NSCLC patients may need more large clinical studies to explain. And there are still many problems with the use of EGFR-TKIs in the perioperative period of stage III patients. The dose and duration of neoadjuvant EGFR-TKIs therapy, as well as the optimal timing of post-treatment surgery, remain unclear. In this case, the patient received osimertinib plus chemotherapy, which responded well and was well tolerated without increased toxicity. This has been demonstrated previously in clinical studies of EGFR-TKIs plus chemotherapy for advanced NSCLC (27–29). However, large-sample clinical trials are still needed to evaluate whether the use of EGFR-TKIs plus chemotherapy in the neoadjuvant phase of stage III patients are safe and improve efficacy. In addition, few studies have explored the safety and feasibility of using EGFR-TKIs neoadjuvant therapy in initially unresectable stage III disease, and current clinical trials (**Supplementary Table 1**) hardly involve initially unresectable stage III disease, so more studies are needed to explore this in the future. Finally, whether to consider the use of EGFR-TKIs for adjuvant therapy is also an important exploration direction for patients who show good response to neoadjuvant therapy with EGFR-TKIs.

In brief, this is the first report of initially unresectable stage III PSC patient who harbor EGFR mutation benefiting from neoadjuvant therapy with osimertinib plus chemotherapy. This case provides new insights into the treatment in PSC patients.

## Data availability statement

The datasets presented in this article are not readily available because of ethical/privacy restrictions. Requests to access the datasets should be directed to the corresponding author.

## Ethics statement

The studies involving human participants were reviewed and approved by the Research Ethics Committee of the Nanfang Hospital, Southern Medical University. The patients/participants provided their written informed consent to participate in this study. Written informed consent was obtained from the individual(s) for the publication of any potentially identifiable images or data included in this article.

## Author contributions

All authors contributed to the article and approved the submitted version.

## Acknowledgments

The authors wish to gratefully acknowledge this patient and his family for allowing us to publish his clinical case.

## Conflict of interest

Authors YZ and MH were employed by 3D Medicines Inc.

The remaining authors declare that the research was conducted in the absence of any commercial or financial relationships that could be construed as a potential conflict of interest.

## Publisher’s note

All claims expressed in this article are solely those of the authors and do not necessarily represent those of their affiliated organizations, or those of the publisher, the editors and the reviewers. Any product that may be evaluated in this article, or claim that may be made by its manufacturer, is not guaranteed or endorsed by the publisher.
