# Supplementary material for: Case report: an initially unresectable stage III pulmonary sarcomatoid carcinoma qith EGFR mutation achieving pathological complete response following neoadjuvant therapy with osimertinib plus chemotherapy
Source: Front Oncol. 2022 Nov 25;12:1033322. doi: 10.3389/fonc.2022.1033322 (PMC9733669; doi:10.3389/fonc.2022.1033322)
Supplement: Supplementary file 1 [file Presentation_1.pptx]

## Slide 1
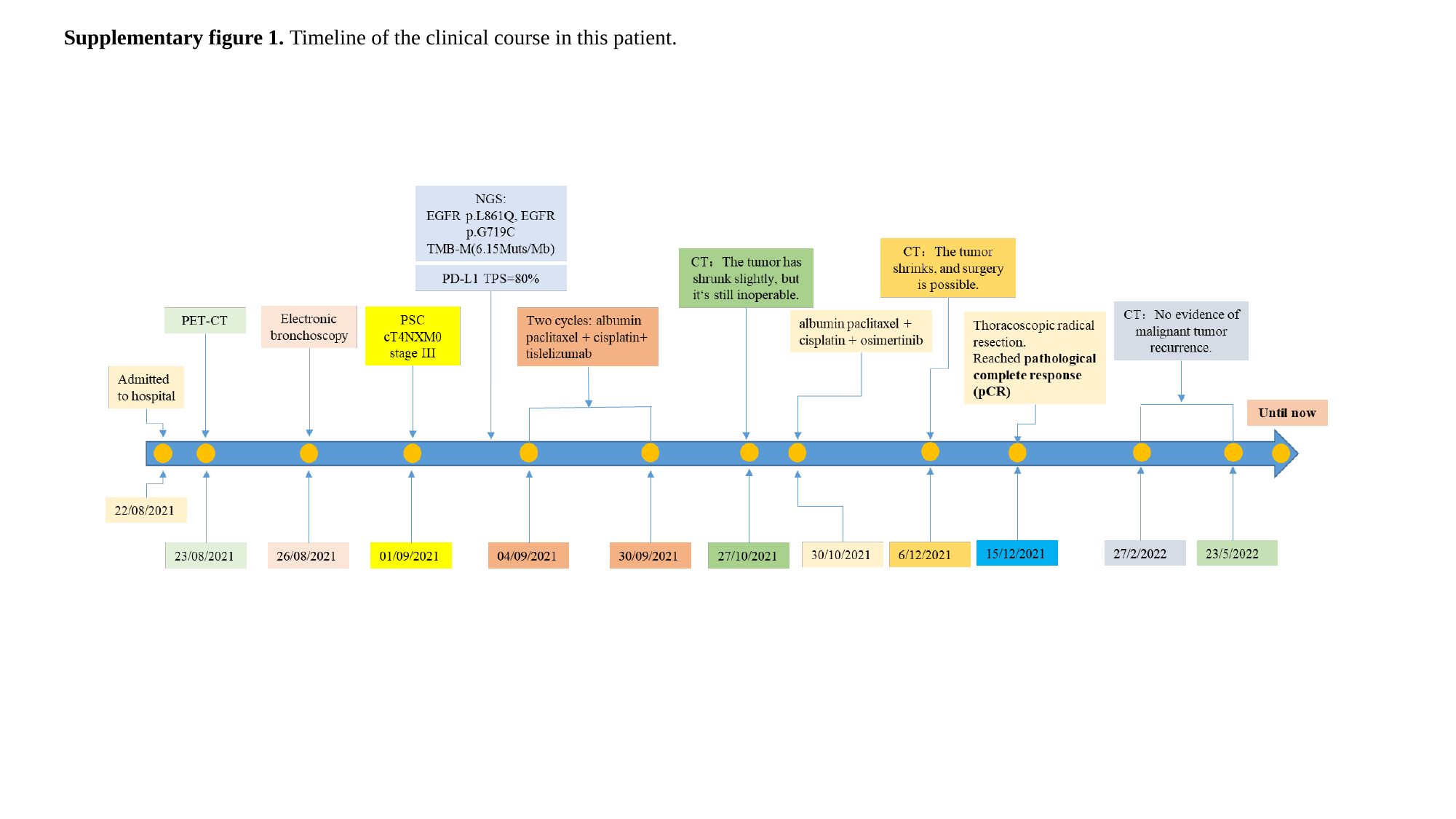

Supplementary figure 1. Timeline of the clinical course in this patient.

## Slide 2
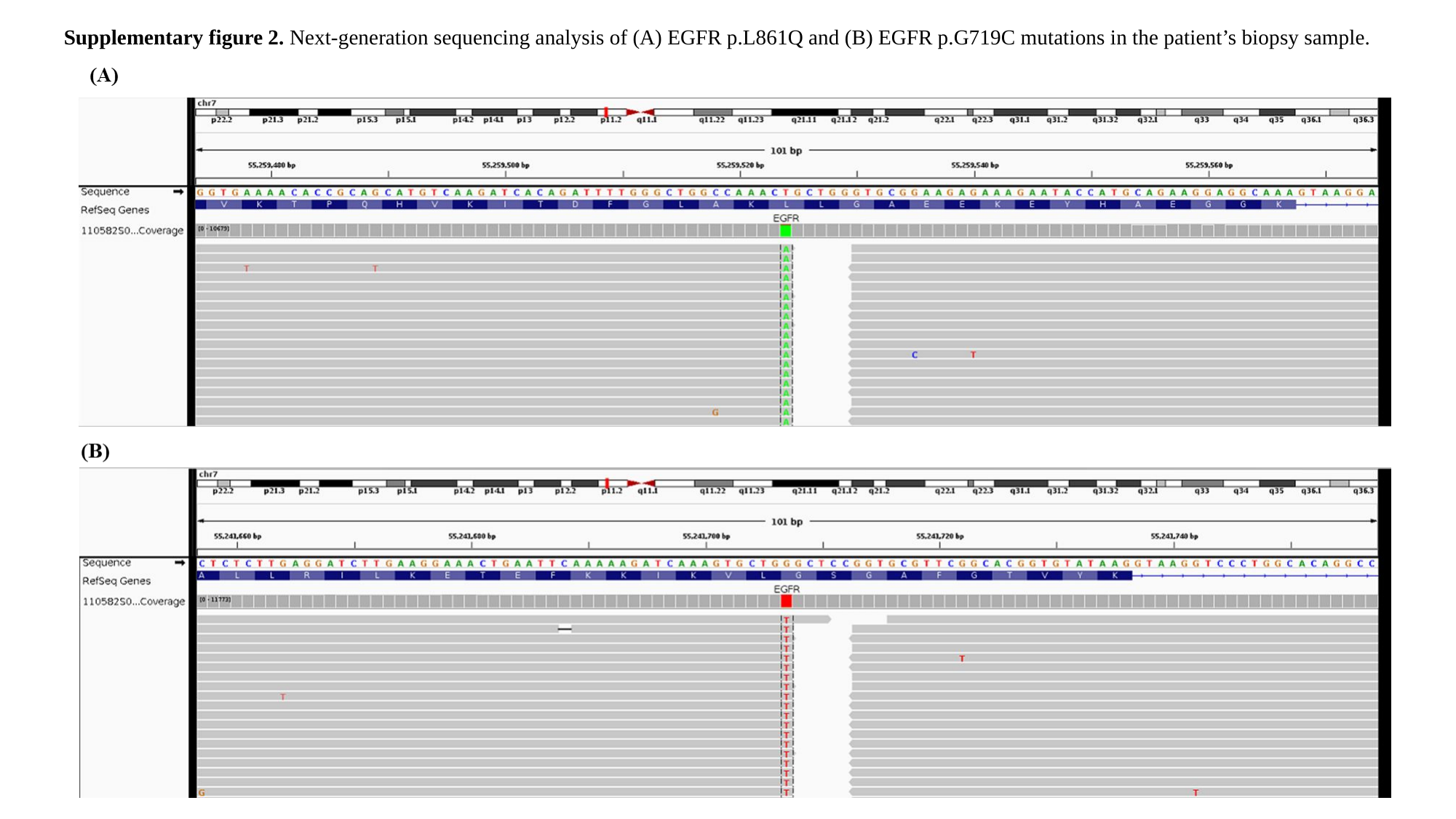

Supplementary figure 2. Next-generation sequencing analysis of (A) EGFR p.L861Q and (B) EGFR p.G719C mutations in the patient’s biopsy sample.
