# Supplementary material for: Case report: an initially unresectable stage III pulmonary sarcomatoid carcinoma qith EGFR mutation achieving pathological complete response following neoadjuvant therapy with osimertinib plus chemotherapy
Source: Front Oncol. 2022 Nov 25;12:1033322. doi: 10.3389/fonc.2022.1033322 (PMC9733669; doi:10.3389/fonc.2022.1033322)
Supplement: Supplementary file 2 [file Table_1.docx]

**Supplementary table 1**. Ongoing clinical trials for EGFR-TKIs in NSCLC neoadjuvant therapy (registered at clinicaltrials.gov).

| NCT number | Study title | Patients |
| --- | --- | --- |
| NCT03749213 | Icotinib as Neoadjuvant Therapy in EGFR-mutant Stage ⅢA-N2 Non-small Cell Lung Cancer | Patients with EGFR-mutant stage ⅢA-N2 NSCLC which considered surgically **resectable** at baseline by the surgical oncologist |
| NCT03349203 | Icotinib as Neoadjuvant and Adjuvant Therapy in EGFR-mutant Stage IIIB or Oligometastasis Non-small Cell Lung Cancer | Patients with EGFR-mutant stage IIIB or oligometastasis NSCLC which **can be potentially radical treated by surgery**. |
| NCT05011487 | Neoadjuvant Osimertinib + Chemotherapy for EGFR-mutant Stage III NSCLC | Patients with **resectable** EGFR-mutant stage III (N2) non-squamous NSCLC. |
| NCT05430802 | Neoadjuvant Furmonertinib and Cisplatin/Pemetrexed as in EGFR Mutated Stage IIIA-IIIB Resectable NSCLC (FORESEE) | Patients with EGFR mutated stage IIIA-IIIB **resectable** NSCLC. |
| NCT05104788 | A Study of Icotinib With Chemotherapy as Neoadjuvant Therapy for Patients With EGFRm Positive Resectable Non-Small Cell Lung Cancer | Patients With EGFR-mutant, **Resectable** for stage II to IIIB (N2) NSCLC. |
| NCT04351555 | A Study of Osimertinib With or Without Chemotherapy Versus Chemotherapy Alone as Neoadjuvant Therapy for Patients With EGFRm Positive Resectable Non-Small Cell Lung Cancer | Patients with completely **resectable** (Stage II - IIIB N2), EGFR-mutant non-squamous NSCLC. |
| NCT03433469 | Osimertinib in Treating Participants With Stage I-IIIA EGFR-mutant Non-small Cell Lung Cancer Before Surgery | Patients with surgically **resectable** stage I-IIIA EGFR-mutant NSCLC. |
| NCT02820116 | The Role of Icotinib in the Perioperative Treatment of IIIA - IIIB NSCLC Patients With EGFR Mutation | Patients with EGFR-mutant **resectable** stage IIIA-IIIB NSCLC. |

ClinicalTrials.gov identifier (NCT number). Information was collected until July 8, 2022.
